# The repertoire of G protein-coupled receptors in the human parasite *Schistosoma mansoni *and the model organism *Schmidtea mediterranea*

**DOI:** 10.1186/1471-2164-12-596

**Published:** 2011-12-06

**Authors:** Mostafa Zamanian, Michael J Kimber, Paul McVeigh, Steve A Carlson, Aaron G Maule, Tim A Day

**Affiliations:** 1 Department of Biomedical Sciences, Iowa State University, Ames, IA USA; 2 Neuroscience Program, Iowa State University, Ames, IA USA; 3 Biomolecular Processes, School of Biological Sciences, Queen's University Belfast, Belfast UK

## Abstract

**Background:**

G protein-coupled receptors (GPCRs) constitute one of the largest groupings of eukaryotic proteins, and represent a particularly lucrative set of pharmaceutical targets. They play an important role in eukaryotic signal transduction and physiology, mediating cellular responses to a diverse range of extracellular stimuli. The phylum Platyhelminthes is of considerable medical and biological importance, housing major pathogens as well as established model organisms. The recent availability of genomic data for the human blood fluke *Schistosoma mansoni *and the model planarian *Schmidtea mediterranea *paves the way for the first comprehensive effort to identify and analyze GPCRs in this important phylum.

**Results:**

Application of a novel transmembrane-oriented approach to receptor mining led to the discovery of 117 *S. mansoni *GPCRs, representing all of the major families; 105 *Rhodopsin*, 2 *Glutamate*, 3 *Adhesion*, 2 *Secretin *and 5 *Frizzled*. Similarly, 418 *Rhodopsin*, 9 *Glutamate*, 21 *Adhesion*, 1 *Secretin *and 11 *Frizzled S. mediterranea *receptors were identified. Among these, we report the identification of novel receptor groupings, including a large and highly-diverged Platyhelminth-specific *Rhodopsin *subfamily, a planarian-specific *Adhesion*-like family, and atypical *Glutamate*-like receptors. Phylogenetic analysis was carried out following extensive gene curation. Support vector machines (SVMs) were trained and used for ligand-based classification of full-length *Rhodopsin *GPCRs, complementing phylogenetic and homology-based classification.

**Conclusions:**

Genome-wide investigation of GPCRs in two platyhelminth genomes reveals an extensive and complex receptor signaling repertoire with many unique features. This work provides important sequence and functional leads for understanding basic flatworm receptor biology, and sheds light on a lucrative set of anthelmintic drug targets.

## Background

The G protein-coupled receptor (GPCR) superfamily constitutes the most expansive family of membrane proteins in the metazoa. These cell-surface receptors play a central role in eukaryotic signal transduction, and conform to a structural archetype consisting of a core domain of seven transmembrane (TM)-spanning *α*-helices. GPCRs are also established drug targets, acting as sites of therapeutic intervention for an estimated 30-50% of marketed pharmaceuticals [1,2]. This is undoubtedly a function of their extensive involvement in a wide range of important physiological processes. The diverse panel of known GPCR ligands includes biogenic amines, photons, peptides, odorants and classical neurotransmitters [3]. This diversity is mirrored by the significant degree of primary sequence variation displayed among GPCRs.

At present, there exists no comprehensive study of GPCRs for the phylum Platyhelminthes. This important phylum houses prominent endoparasites, both flukes and tapeworms, as well as free-living species that serve as established model organisms in the realm of developmental biology. Lack of sequence data and a reliance on techniques with a definably narrow expectation of success such as degenerate PCR have contributed to the very modest number of GPCRs thus far identified or characterized [4-9] in this phylum. The arrival of EST repositories [10-12] has only marginally contributed to this number, perhaps as a consequence of GPCR under-representation [13]. The recent availability of *Schistosoma mansoni *[14] and *Schmidtea mediterranea *[15] whole genome sequence data provides basis for the *in silico *accumulation and analysis of undiscovered and potentially novel receptors.

The blood fluke *Schistosoma mansoni *is the primary etiological agent of human schistosomiasis, a chronic and debilitating condition that afflicts a staggering 207 million people in 76 countries [16] and accounts for 280,000 deaths per annum in sub-Saharan Africa alone [17]. It is calculated that up to 70 million disability-adjusted life years (DALYs) are lost to schistosomiasis annually [18]. This figure surpasses the global burden posed by both malaria and tuberculosis, and is nearly equivalent to that of HIV/AIDS. At present, this overwhelming disease burden is met with a near exclusive reliance on treatment with the drug praziquantel. The threat of drug resistance [19,20] has spurred recognition of the pressing need for new antischistosomals [21-23]. In this context, as modulators of a diverse range of critical biochemical and physiological pathways, GPCRs hold great promise as potential targets for disruption of crucial parasite survival and proliferation activities.

The free-living planarian *Schmidtea mediterranea *is an important platyhelminth studied extensively for its regenerative abilities [24,25]. Like other planarians, it is abundantly seeded with totipotent stem cells with the ability to migrate and undergo division and differentiation at sites of injury. In addition to its current role as a powerful model organism for regeneration and stem cell biology, *S. mediterranea *presents itself as a potential parasite drug discovery model [26]. In the case of nematodes, the biology of the free-living model organism *Ceanhorhabditis elegans *features prominently in many anti-parasitic drug discovery efforts [27,28]. Like *C. elegans*, *S. mediterranea *is significantly more tractable to modern genomic approaches compared to the parasitic members of its phyla. It is relatively easy to maintain and it is amenable to RNA interference (RNAi) [29]. Genome-wide analysis and comparison of the GPCR complements of *S. mansoni *and *S. mediterranea *is a major step towards engaging this hypothesis.

The growing number of sequenced genomes has provided a GPCR mining platform for a number of organisms, including *Homo sapien *[30], *Mus musculus *[31], *Gallus gallus *[32], *Rattus rattus *[33], *Tetraodon nigrovirdis *[34], *Anopheles gambiae *[35], *Drosophila melanogaster *[36], *Ciona intestinalis *[37], *Branchiostoma floridae *[38], *Xenopus tropicalis *[39] and *Canis familiaris *[40]. For these organisms, GPCR sequences have been accumulated with a range of bioinformatic methods that include homology-based searching (BLAST), hidden Markov models (HMMs) and motif-driven queries [41]. The more sophisticated GPCR mining protocols have involved the application of a combination of such methods and algorithms.

Phylogenetic studies of the GPCRs in a number of eukaryotic genomes have led to the introduction of the GRAFS classification system [42,43]. GRAFS outlines five major protein families thought to represent groupings of receptors with shared evolutionary ancestry present in the Bilateria: *Glutamate*, *Rhodopsin*, *Adhesion*, *Frizzled*, and *Secretin*. In addition to these primary families, some organisms are known to house groupings of lineage-specific receptors that constitute distinct GPCR families. Examples in the phylogenetic vicinity of the Platyhelminthes include the nematode chemosensory receptors [44] and insect gustatory receptors [45]. Any *in silico *protocol for genome wide GPCR identification should therefore cast a broad enough net to reveal any such highly-diverged receptor groupings, while also providing stringency to limit false positives.

Here, we apply an array of sensitive methods towards the goal of identifying, manually curating and classifying putative G protein-coupled receptor sequences in two prominent platyhelminths. Our hypothesis is that organisms in this phylum possess an extensive and complex complement of GPCRs, including phylum or species-specific GPCR groupings. We perform phylogenetic analysis of putative receptors with respect to the GRAFS classification system and employ a machine-learning approach for ligand-based classification of full-length *Rhodopsin *GPCRs.

## Results and Discussion

**Figure 1:**
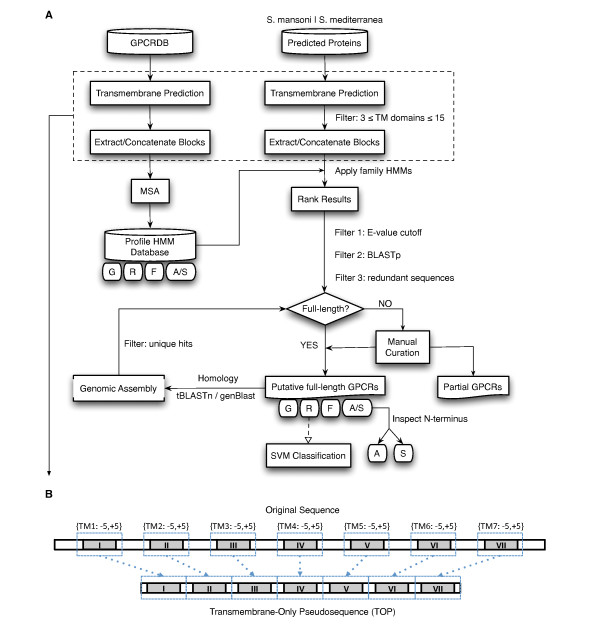
**Transmembrane domain-focused GPCR sequence mining strategy**. (A) Family-specific profile HMMs are built using TM-only pseudosequences (TOPs) extracted from the GPCRDB [49] sequence repository. The predicted proteomes of both *S. mansoni *and *S. mediterranea *are processed in a manner identical to that of the training sequences and are searched against a set of family-specific profile HMMs. Results are ranked statistically and sequences meeting a conservatively selected cutoff undergo an automated BLASTp campaign against the NCBI "nr" database. The output is parsed, and transmembrane proteins exhibiting significant homology to non-GPCR proteins are removed. Redundant sequences are removed with the BLAT utility. The surviving sequence pool is then manually assessed and curated, followed by homology-based searches of these sequences against the whole genome assemblies. *Adhesion *and *Secretin *GPCR sequences are distinguished from one another by inspection of their N-terminal ectodomains. Putative full-length *Rhodopsin *GPCRs, defined by the presence of an intact 7TM domain, are sub-classified via SVM. (B) Construction of TOPs is a two-step process involving the prediction of TM boundary coordinates by HMMTOP, followed by the ordered concatenation of TM domains flanked bi-directionally by 5 amino acids.

In this study, we developed a robust transmembrane-focused strategy to identify, curate and classify putative platyhelminth GPCRs. TM-focused profile hidden Markov models (HMMs) were used to mine the predicted proteomes of *S. mansoni *and *S. mediterranea *in order to identify receptors at the GPCR family plane. Subsequent rounds of filtering were used to remove false positives, followed by homology-based searches against the original genome assemblies. Extensive manual curation of the final sequence dataset allowed for more refined phylogenetic analysis. Greater classification depth was achieved with a complementary transmembrane-focused support vector machine (SVM)-based classifier. An overview of this bioinformatics protocol is outlined in Figure 1.

### Identification of GRAFS family receptors with TM-focused profile HMMs

Towards the goal of identifying members of the GRAFS GPCR families in our genomes of interest, we relied primarily on the use of family-specific profile HMMs. This alignment-rooted method has been successfully applied in other genomes and has been shown suitable for the identification and classification of GPCR sequences at the family level [41,46]. In a departure from previously described protocols, we chose to focus HMM training exclusively on the most highly-conserved structural features that extend throughout the GPCR superfamily. The idea behind this measure was to dampen challenges posed by the inexact gene structures that underlie the flatworm predicted proteomes, as well as the sizable phylogenetic distance of this phylum from organisms with characterized GPCR complements.

In this framework, receptor transmembrane domains are convenient markers that can be identified with greater confidence than other GPCR stretches using sensitive prediction algorithms such as HMMTOP [47] and TMHMM [48]. Training sequences were procured from GPCRDB [49] and processed into what we will refer to as "transmembrane-only pseudosequences" (TOPs), representing the ordered concatenation of TM domains flanked bi-directionally by 5 amino acids (Figure 1b). TM-focused HMMs were constructed for each of the major GPCR families, as well as for the nematode chemosensory and insect odorant families. The *Adhesion *and *Secretin *training sets were combined to build a single HMM, given that sequences belonging to these families are not easily distinguishable beyond the N-terminal ectodomain [13].

The predicted proteomes of *S. mansoni *and *S. meditearranea *were first filtered for the removal of globular proteins. Typical strategies limit investigation to proteins with 6-8 predicted TM domains, tolerating errors in the algorithmic prediction of these regions. We significantly relaxed this filter and broadened the search scope to include all proteins with 3-15 TM domains. The utility of this change then was to alert us to partial sequences or incorrectly predicted gene models that may be reconstructed with manual curation and that otherwise would have been screened from detection. Family-derived profile HMMs already provide an adequately stringent filter for distinguishing between GRAFS family receptors and other transmembrane proteins.

The proteins that survived this filter were processed into TOPs in the same manner as the training sequences. These sequences were searched against the set of profile HMMs, and the resulting hits for each GPCR family were ranked according to E-value. A primary cut-off was selected at the point where subsequent hits showed significant homology to other known proteins or GPCRs belonging to other families. This was accomplished with a BLASTp [50] search of all hits against the NCBI non-redundant (nr) database (Additional File 1). Sequences that displayed GPCR-related homology, along with those that returned no significant BLAST results, were retained. As evidenced later, the requirement of statistically meaningful GPCR-related homology introduces an unnecessary selection bias that can mask the identification of unique receptors.

**Figure 2:**
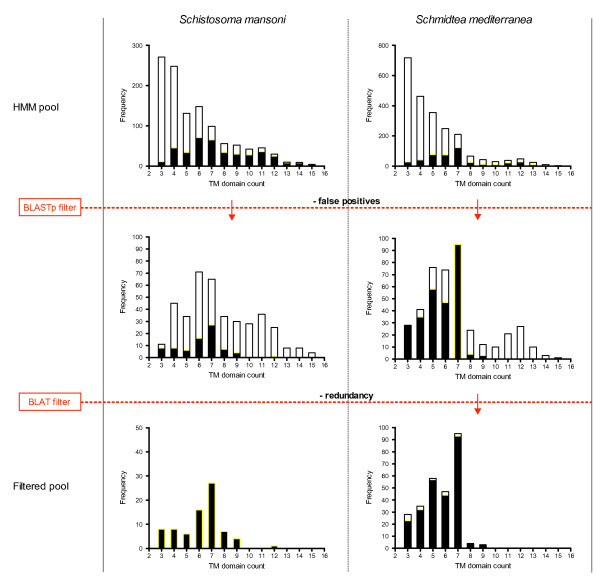
**HMM-based identification of *S. mansoni *and *S. mediterranea *GPCRs**. The transmembrane frequency distribution of the *S. mansoni *(left) and *S. mediterranea *(right) predicted proteomes is shown as predicted by HMMTOP at various junctures of the bioinformatics protocol for the *Rhodopsin *family. The top graphs overlay the HMM-derived sequence pools (black, yellow outline) on top of the entire predicted proteomes (white, black outline) in the assayed TM domain range (3-15). The middle graphs overlay the BLASTp filtered sequence pools (black, yellow outline) on top of the HMM-derived pools (white, black outline). The bottom graphs display the final distributions upon filtering, and after the removal of redundant sequences in the case of *S. mediterranea*.

Application of the *Rhodopsin *HMM to the *S. mansoni *predicted proteome led to the examination of the 400 top-ranking hits (E-value < 0.007), 77 of which remained after removal of false positives via homology-based searches. Similarly, 270 of the 450 top-ranked (E-value < 0.002) *Rhodopsin *HMM hits remained for *S. mediterranea*. Redundancy within the *S. mediterranea *genome assembly warranted the detection and removal of identical sequences. BLAT [51] was used to self-align the nucleotide sequences of the predicted proteins that survived the HMM filtering process. Redundant sequences were removed and if a choice was presented, the longest member of a set of identical sequences was retained. This led to the removal of 14 *Rhodopsin *sequences from the *S. mediterranea *dataset. Figure 2 displays the overall transmembrane distribution for both proteomes at these various stages of processing for the *Rhodopsin *family. These steps were likewise performed for the nematode chemosensory and insect odorant GPCR families, however no flatworm orthologs were identified. This is not unexpected, considering their lack of conservation among the Ecdysozoa.

### Manual editing of gene models

**Figure 3:**
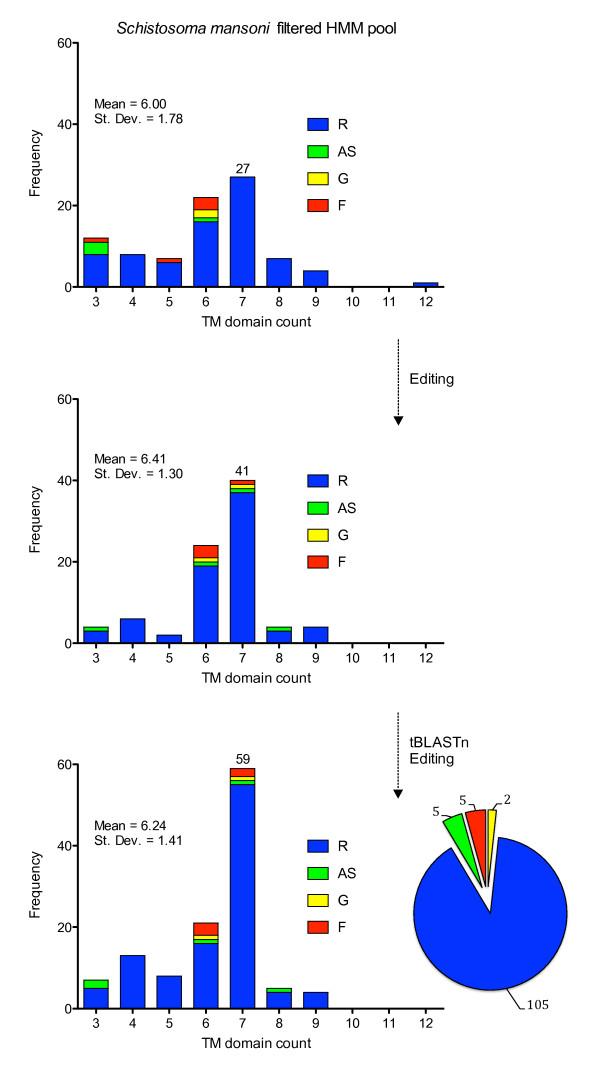
**Manual curation and expansion of *Schistosoma mansoni *GRAFS GPCRs**. The transmembrane distributions for the filtered *S. mansoni *HMM pool is shown before (top) and after (middle) manual editing of the underlying gene models, as predicted by HMMTOP. The number of GPCRs with a predicted intact 7TM domain increases from 27 to 41, coupled with a significant contraction of the distribution spread. The mean TM count shifts from 6.00 to 6.41, which equates to the identification and addition of roughly 42 missing TM domains during the first round of curation. Homology-bases searches against the genome assembly increased the putative 7TM receptor count to 59 (bottom). Receptors in the 8 and 9 TM bin can be considered full-length for our purposes, as the erroneously-predicted additional TM domains can be excised for phylogenetic analysis. Inclusion of these receptors brings the total putative full-length (7TM) receptor tally to 68 (of 117 total sequences).

Candidate GPCR sequences underwent manual inspection, and the corresponding gene models were edited where necessary. This labor-intensive step is crucial in improving the reliability of any further analysis on this gene family. Common manual edits included the merging or splitting of gene models, movement of intron-exon boundaries, and sequence extension or truncation. This process was aided by examination of open reading frames (ORFs) in the vicinity of a gene models. ORFs that housed common receptor motifs, displayed GPCR-related homology or contained transmembrane stretches were typically incorporated. In many cases, sequencing gaps prevented any meaningful improvement. *S. mansoni *GRAFS sequences and *S. mediterranea *GAFS sequences were curated in this manner. We avoided genome-wide manual curation of *S. mediterranea Rhodopsin *sequences in light of the dubious condition of the draft genome. The A/T rich (69%), highly repetitious (46%) and heterozygous nature of the genome has complicated automated assembly efforts. However, as we elaborate later, we did construct and edit gene models for a particular grouping of *Rhodopsin*-like planarian GPCRs. The significant level of improvement achieved by manual gene editing is shown in Figure 3.

### Homology-based expansion and final gene editing

To account for the likelihood that the primary sets of gene models used do not provide perfect accounting of all gene-encoding regions within the assemblies, we exercised a translated nucleotide BLAST (tBLASTn) and carried out genome-wide gene prediction using genBlastG [52]. For each receptor family, putative full-length GPCRs were combined from both species and used in parallel to query the nucleotide assemblies and to generate homology-based gene models. tBLASTn hits with E-value < 0.1 were examined for GPCR-related homology, and genBlast prediction was carried out with default settings (E-value < 0.01). In cases where identified regions of homology overlapped with a given gene model, that gene model was added to the sequence pool. Conversely, if no gene model was found to be present at a particular genomic location, a simple preliminary gene model was built by connecting the high-scoring segment pairs (HSP) that contributed to the hit. In keeping with the HMM approach, only putative receptors with a TM count ≥ 3 were retained. CD-HIT [53] was used at 90% sequence identity to generate a non-redundant dataset and to help remove splice variants, polymorphisms and previously identified genes.

This led to a further significant expansion of the total unique sequence count in both organisms (Table 1). This reported sequence count is not equivalent to a receptor count, as many of these sequences may represent fragments of a single protein or prove to be redundant sequences. To bridge this gap and to improve the general state of this additional sequence data, manual editing of gene models was again performed. Comparison of our final receptor dataset to GPCRs uncovered in the recently released Smed454 transcriptome dataset [54] is encouraging. Of 79 receptors identified, only five short-length sequences (< 100 bp) were found absent from our dataset. Fragments of these sequences were found in the assembly, but were filtered due to their lengths. With subsequent improvements in the assembly, we could attempt to 'rescue' these and similar sequences.

**Table 1:** Tabulated GPCR sequence count at various stages of processing

	*S. mansoni*				*S.mediterranea*					
**Family**	**HMM**	**MC**	**H**	**Final**	**HMM**	**R**	**MC**	**H**	**R**	**Final**

**G**	2	2	3	**2**	6	6	6	10	9	**9**
**R**	77	74	105	**105**	270	256	-	291	418	**418**
**AS**	4	4	5	**5**	11	11	11	30	24	**22**
**F**	5	4	5	**5**	10	8	8	11	10	**11**

Sequence counts are provided for each GPCR family at different stages in the receptor mining protocol. The *S. mansoni *count is shown after application of the TM-focused HMM and filtering of the predicted proteome (HMM), extensive manual curation (MC), homology-based searches against the nucleotide assembly (H) and a final round of manual curation (Final). This progression is similarly displayed for *S. mediterranea*, with additional stages for the shedding of redundant sequences using BLAT (R).

### Overall phylogenetic view

**Figure 4:**
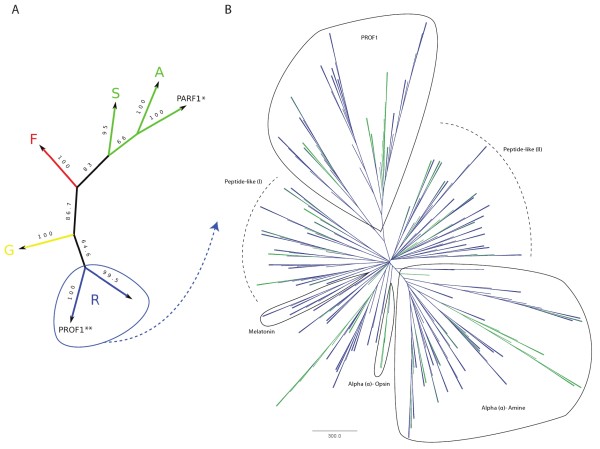
**GRAFS and *Rhodopsin *phylogenetic trees**. (A) Overall topological view of the combined *S.mansoni *and *S. mediterranea *GPCR complements. Maximum parsimony analysis (bootstrap value = 100) was carried out using putative full-length non-*Rhodopsin *GPCRs and a subset of full-length *Rhodopsin*-like GPCRs. In addition to the phylogenetic clustering of sequences into the primary GRAFS families, this analysis reveals the presence of two distinct phylum-specific groupings: PROF1 and PARF1. * Sequence family is present in *S. mediterranea*. ** Sequence family is present in both *S. mansoni *and *S. mediterranea*. (B) Neighbor-joining tree of flatworm *Rhodopsin*-like GPCRs. To maximize the number of sequences included in this analysis, a sequence block housing TM domains I-IV was extracted from the overall alignment. This allowed for inclusion of 312 *Rhodopsin*-like sequences: 90 *S. mansoni *and 224 *S. mediterranea *receptors (bootstrap value = 200). The amine, opsin, peptide, melatonin, and PROF1 groupings are highlighted. Branches terminating in *S. mansoni *receptors are shown in green, and branches terminating in *S. mediterranea *receptors are shown in blue.

Putative receptor sequences were tentatively divided into three sequence bins based on the number of predicted TM domains: full-length, near full-length and partial. Full-length sequences were those that likely had their entire 7TM domain intact as predicted by HMMTOP with user oversight. Alignments to homologous proteins were used to help make a final decision with respect to the potential algorithmic miscounting of TM domains. Near full-length sequences are predicted to contain ≥ 4 TM domains, while all other sequences (< 4 TM domains) were placed into the partial sequence bin. Phylogenetic analysis was carried out for full-length and many near full-length receptors. Figure 4a displays a topological overview of the primary flatworm GPCR groupings. This phylogenetic analysis confirms the distinct and analogous presence of the primary GRAFS families, and further reveals two novel flatworm GPCR families: Platyhelminth-specific *Rhodopsin*-like orphan family 1 (PROF1) and Planarian *Adhesion*-like receptor family 1 (PARF1).

### The *Rhodopsin *family

The *Rhodopsin *family is divided into four main groups (*α*, *β*, *δ*, and *γ*) and further subdivided into 13 major sub-families via analysis of fully sequenced mammalian genomes [55]. The *α *and *β *subfamilies are well-populated in both *S. mansoni *and *S. mediterranea *(Figure 4b), while the *δ *and *γ *subfamilies are absent. Table 2 provides a preliminary classification of receptors identified with respect to the GRAFS classification system from a comparative perspective.

**Table 2:** GRAFS-based comparison of GPCR repertoires

		*H. sapiens*	*A. gambiae*	*D. melanogaster*	*C. elegans*	*S. mansoni*	*S.mediterranea*
**R**	AMIN (*α*)	44	18	21	20	24	66
	MEC (*α*)	22	2	1	1	0	0
	MTN (*α*)	3	2	2	0	0	9
	OPN (*α*)	11	12	8	1	4	7
	PTGER (*α*)	13	0	0	0	0	0
	PEP (*β*)	43	29	21	31	36	130
	CHEM (*γ*)	43	0	0	0	0	0
	MCHR (*γ*)	1	0	0	0	0	0
	SOG (*γ*)	10	3	5	10	0	0
	LGR (*δ*)	7	3	4	1	0	0
	MRG (*δ*)	7	0	0	0	0	0
	OLF (*δ*)	494	0	0	0	0	0
	PUR (*δ*)	35	0	0	0	0	0
	PROF1	0	0	0	0	19	47
	Unclassified	20	77	79	124	22	159

**F**	FZD/SMO	11	13	5	5	5	11
	TAS2	13	0	0	0	0	0

**G**	GLR	24	8	9	6	2	9

**A/S**	ADH	27	13	5	5	3	9
	SEC	20	1	13	5	2	1
	PARF1	0	0	0	0	0	12

The GPCR repertoires of *S. mansoni *and *S. mediterranea *are shown from a GRAFS-based perspective, alongside those of other organisms with characterized GPCR complements. For *Rhodopsin *sub-classification, BLAST searches were used to help tentatively assign putative ligands to receptors omitted from phylogenetic analysis.

#### Alpha (α) receptors

**Figure 5:**
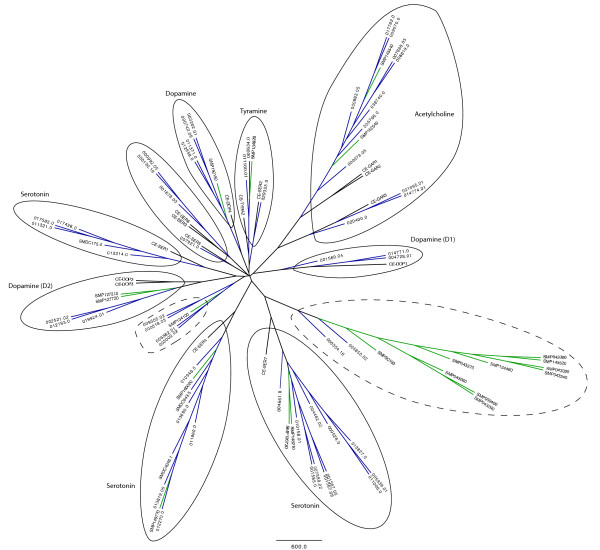
**Aminergic receptors: *S. mediterranea *and *S. mansoni***. Neighbor-joining tree (bootstrap value = 500) of putative aminergic GPCRs. Included in this analysis are 21 (of 24) *S. mansoni *and 57 (of 66) *S. mediterranea *full-length and near full-length aminergic receptors, alongside 14 known *C. elegans *amine receptors. The latter grouping includes receptors that respond to tyramine, octopamine, dopamine, serotonin, and acetylcholine [97]. Branch lengths are scaled to bootstrap support, branches terminating in *Schistosoma *receptors are shown in green, and branches terminating in *Schmidtea *receptors are shown in blue. Flatworm receptors are outlined (solid lines) and classified by ligand with respect to their nearest-related *C. elegans *homologs. Two diverged flatworm-specific receptor groupings are outlined with a dashed line.

The *α *subfamily houses amine, opsin-like, and melatonin receptors. Among these, the amine grouping is typically largest. This metazoan trend holds true for *S. mansoni *and *S. mediterranea*, each possessing at least 24 and 66 putative aminergic receptors, respectively. These numbers are greater than those observed among ecdysozoans, and in the case of *S. mediterranea*, the figure surpasses even the human amine GPCR complement. Biogenic amines such as serotonin (5-hydroxytryptamine; 5HT), dopamine, and histamine have been shown to play a prominent role in the flatworm nervous system [56,57]. Although a small number of aminergic GPCRs have been characterized in this phylum, the majority of receptors that mediate aminergic signaling have thus far remained elusive. Phylogenetic analysis was carried out on the flatworm amine GPCR complement with respect to *C. elegans *aminergic receptors, as shown in Figure 5. Two diverged flatworm-specific groupings can be outlined, including one that signifies a major paralogous expansion in schistosomes. Other flatworm receptors are grouped and tentatively associated with ligands corresponding to their phylogenetic relationships with deorphanized *C. elegans *GPCRs.

Four melanopsin-like receptors were identified in *S. mansoni*. Six melanopsin-like receptors were identified in *S. mediterranea*, along with a single receptor that displays moderate homology to various ciliary opsins. Along with the presence of cyclic nucleotide gated (CNG) ion channels in the planarian genome, this raises the possibility of ciliary phototransduction. Another noteworthy observation is the conspicuous absence of melatonin-like receptors in *S. mansoni*, while *S. mediterranea *houses a relatively large complement of 9 such receptors. Melatonin is endogenously synthesized in planaria in a circadian manner [58,59], and has been implicated in regeneration [60]. Identification of melatonin receptors is a requisite for a more complete mapping of the underlying signal transduction pathway(s) in these important processes.

#### Beta (β) receptors

The *β *subfamily contains the great majority of peptide and peptide hormone GPCRs in examined organisms. Neuropeptidergic signaling is known to play a fundamental role in flatworm locomation, reproduction, feeding, host-finding and regeneration [61,62]. The known flatworm neuropeptide complement has recently undergone considerable expansion with the application of bioinformatics and mass spectrometry-based (proteomics) approaches [63,64]. This represents a significant advance from the original handful of FMRFamide-like peptides (FLPs) and neuropeptide Fs (NPFs) first identified in the phylum. Many of these newly-identified amidated peptides are planarian or flatworm-specific, while others exhibit relatedness to peptides in other phyla, including myomodulin-like, buccalin-like, pyrokinin-like, neuropeptide FF (NPFF)-like, and gonadotropin (or thyrotropin) releasing hormone-like peptides.

Our efforts yielded at least 130 and 36 putative peptide receptors in *S. mediterranea *and *S. mansoni*, respectively. These numbers further evidence the notion that peptidergic signaling is the predominant mode of neurotransmission in the Platyhelminthes. Flatworm peptide receptors do not cluster into a single phylogenetic group (Figure 4b). It can be noted that the putative flatworm peptide receptor count greatly outnumbers the set of currently known peptide ligands. Although this may be explained by peptide promiscuity and receptor redundancy, it is also very possible that many neuropeptides have yet to be uncovered. Ligands cannot be confidently assigned to the majority of identified receptors. While some show moderate homology to characterized FLP and NPF-like receptors, most receptors display weak or insignificant homology to an assortment of thyrotropin-releasing hormone, capa, sex peptide, growth hormone secretagogue, proctolin, pyrokinin, myokinin, tachykinin, galanin, and orexin receptors. These tentative BLAST-based annotations (Additional File 2) may be used with caution to help guide receptor deorphanization efforts.

#### Unclassified receptors

A large number of *Rhodopsin *receptors could not be individually annotated with confidence, and were placed in the "Unclassified" *Rhodopsin *receptor bin. Receptors in this category lack phylogenetic support to be clustered with known *Rhodopsin *groupings and lack meaningful homology to receptors with known ligands. Many receptors in this bin exhibit some weak peptide or amine receptor-relatedness, but these require functional validation before they can be added to the *α *or *β *subfamily counts. Many of these receptors are likely unique to the phylum, and therefore obscure the *Rhodopsin *family subdivisions apparent in the vertebrate lineage.

#### Planarian homologs of parasite GPCRs

Given the relative tractability of planarians to experimental manipulation, we identified the nearest homologs of *S. mansoni Rhodopsin *receptors in the *S. mediterranea *pool (Additional File 3, Table S1). It is a reasonable expectation that there is significant conservation in the biological and pharmacological properties of receptors sharing high sequence identity between these species. The characterization of certain planarian receptors is likely to inform us about the function and druggability of parasite receptors. Each *S. mansoni *receptor was first matched to its most similar *S. mediterranea *sequelog, and sequence pairs were ranked according to amino acid percent identity (PID): 6 receptor pairs were identified sharing > 50% PID, 21 with 40-50% PID, 48 with 30-40% PID, and the remaining sequences with < 20% PID. The top grouping is comprised exclusively of amine (GAR and 5HT) and peptide GPCRs. Among them is a receptor pair orthologous to Gt-NPR1 [7], the only neuropeptide receptor deorphanized in this phylum. This degree of sequence conservation promotes the use of planaria as a convenient heterologous system to study parasite receptors.

### Platyhelminth-specific *Rhodopsin*-like orphan family 1 (PROF1)

A large and distinct sequence clade comprised of 19 *S. mansoni *and 47 *S. mediterranea *proteins was identified and labeled Platyhelminth *Rhodopsin *Orphan Family 1 (PROF1). Members of this novel and highly-diverged phylogenetic grouping are predicted to house a 7TM domain with an extracellular N-terminus and seem to be exclusively derived from intronless genes. Most PROF1 sequences were revealed with homology-based searches after a small number of bait sequences survived the *Rhodopsin *HMM filtering stage. In fact, 38 of the 47 *S. mediterranea *PROF1-containing ORFs were identified via homology-based searches, and only one of these ORFs coincided with an existing gene model. Similarly, 13 of 19 *S. mansoni *PROF1 were identified in this manner and only four of these were represented in the predicted gene set.

These receptors display remnants of classical *Rhodopsin *motifs at corresponding positions (Table 3), yet show no significant overall homology to any previously discovered GPCRs. It is valuable to point out that the absolute requirement of GPCR-related BLAST homology as part of the post-HMM filtering stage would have masked the identification of PROF1 receptors. BLASTp searches of all PROF1 sequences against the NCBI nr database (E-value cutoff = 0.1) returned no hits for the majority of sequences. The small pool of hits that did result, exhibit both very poor homology and represent an incongruous range of receptors that include peptide, lipid and odorant GPCRs. This further highlights the unique nature of these receptors.

**Table 3:** Comparison of PROF1 motifs and classical *Rhodopsin *motifs

Location	*Rhodopsin*	PROF1-I	PROF1-II	PROF1-III
TM2	LA..D	[**L**/**I**][**A**/**S**]..[**D**/**E**]	[**L/I**][**A/T**]..[H/N]	[**L/I**]**A**..[**D/E**]
TM3/IL2	[D/E]R[Y/H]	**D**[**R/K**][C/M]	**DR**[L/V]	**D**[**R/S**]C
TM6	[F/Y]...W.P	[**F/Y**]...[S/A].[**P/L**]	[**F/V**]...T.**P**	S...I.S
TM7	[N/D]P..Y	[**N/S**][F/G]..[**Y/F**]	[**N/D**]F..[**Y**/**F**]	**N**[F/I]..[M/L]

PROF1 motifs are compared to ubiquitous *Rhodopsin *family motifs. Motifs are displayed as regular expressions. The two most frequently occurring amino acids are shown for each position in order of frequency, except in cases where a particular residue is absolutely conserved or when there is no clear second in frequency rank. Bolded text is used to highlight positional agreement between PROF1 motifs and the corresponding *Rhodopsin *motifs. More specifically, instances where the most frequently occurring residue is equivalent.

**Figure 6:**
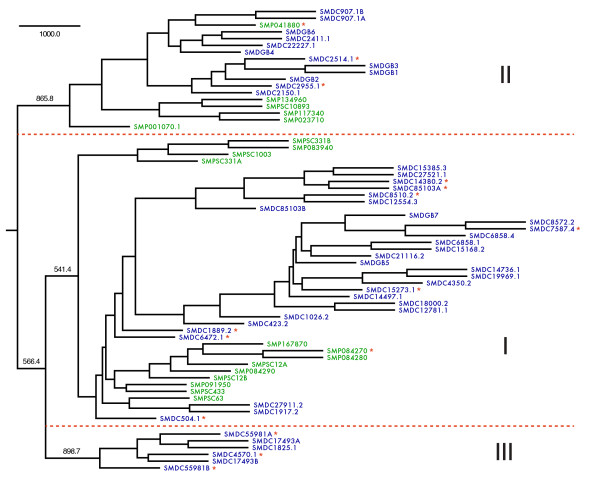
**Phylogenetic analysis of PROF1 GPCRs**. Maximum parsimony tree for all identified PROF1 receptors. An alignment block that included TM domains I-IV was bootstrapped 1000 times for parsimony analysis. PROF1 can be subdivided into 3 families with good bootstrap support (> 50%; relevant values displayed): I, II and III. *Schistosoma *sequences are shown in green and *Schmidtea *sequences are shown in blue. The tree is rooted with a schistosome opsin-like GPCR (AAF73286.1). *Schmidtea *PROF1 receptors with transcript expression confirmed by RT-PCR are marked with red asterisks.

**Figure 7:**
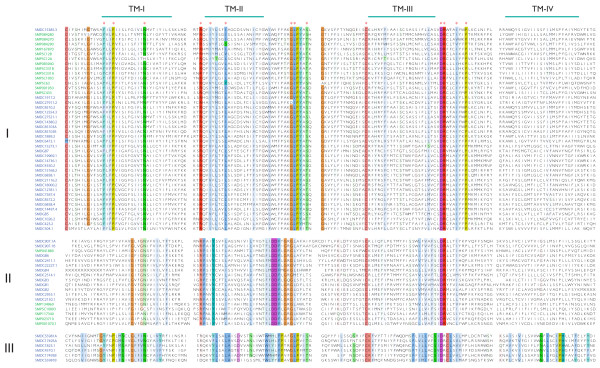
**PROF1 multiple sequence alignment**. Multiple sequence alignment of all PROF1 receptors over a sequence range that includes TM domains I-IV (used for phylogenetic analysis). Residues are colored according to a conservation threshold set at 80% within each group. The locations of individual TM domains were approximated by alignment to Rhodopsin and are depicted above the MSA. Red asterisks are used to mark residue locations where the among-group PROF1 identity level threshold (> 80%) is met.

Maximum parsimony analysis led to the subdivision of PROF1 into three primary phylogenetic groupings with good bootstrap support (Figure 6). Group I is the largest among these with 29 and 13 members from *S. mediterranea *and *S. mansoni*, respectively. The lack of obvious one-to-one orthologs between species suggests expansion or contraction of these receptors occurred after the splitting of planaria and trematodes in the flatworm lineage. Group II includes 6 *S. mansoni *and 12 *S. mediterranea *sequences, while group III houses 6 *S. mediterranea *sequences. It is likely that the closest related receptor to the ancestral gene for this family is contained in group I or II. A multiple sequence alignment of TM domains I-IV (used for phylogenetic analysis) is shown in Figure 7.

Of additional interest, short PROF1-like sequence fragments were identified in both genome assemblies that could not be incorporated into full-length gene structures. These may constitute pseudogenes, or be ascribed to errors in assembly. RT-PCR was used to confirm transcript expression for a selection of putative full-length planarian PROF1 receptors: 8 from group I, 2 from group II and 3 from group III (highlighted in Figure 6). Correct-sized amplicons were visualized for all 13 targets. Similarly, we selected a representative from each schistosome PROF1 grouping and confirmed transcription in the adult stage. It is not currently possible to assign functions or putative ligands for the PROF1 family. However, given that they constitute one of the largest *Rhodopsin*-like subfamilies conserved between these monophyletic species, we suspect that they play an important biological role in this phyla.

### The *Adhesion *and *Secretin *Families

*Adhesion *and *Secretin *receptors share sequence similarity in their 7TM domains and are commonly grouped as Class II GPCRs. The phylogenetic separation of these families under the GRAFS paradigm is mirrored by noticeable structural differences in their N-terminal ectodomains. Archetypal *Adhesion *GPCRs have a long N-terminus containing a diverse arrangement of functional domains. In the vertebrate lineage, this family constitutes the second largest grouping of GPCRs after *Rhodopsin *and is further partitioned into eight clusters. *Secretin *GPCRs usually display N-terminal hormone-binding domains (HBD) that confer responsivity to peptide hormones and are thought to descend from the group V *Adhesion *receptors [65]. Additional *Adhesion*-like proteins have been identified in various organisms that stake more dubious evolutionary positions. The insect *Methuselah *receptors are one such example that have become the subject of great investigation, attributable to their role in life-span regulation and stress resistance [66]. More recently, a cluster of *Adhesion*-like receptors (NvX) was identified in the basal cnidarian *N. vectensis *which share some homology with *Methuselah *receptors [65,67].

We have identified a novel cluster of 12 *Schmidtea *GPCRs that show moderate (> 20% PID) homology to NvX receptors. We denote this cluster Planarian *Adhesion*-like receptor family 1 (PARF1). Like NvX, PARF1 receptors contain a single Somatomedin B domain, except in the case of SMDC005966C which is predicted to contain two. Interestingly, no schistosome PARF1 orthologs were identified. A single *Adhesion *GPCR in *S. mansoni *(SMP099670) was found to house a Somatomedin B domain, but it otherwise shares no significant homology with planarian PARF1 receptors. Two *Adhesion*-like *Schmidtea *GPCRs (SMD002396 and SMD002965) were identified that most resemble vertebrate group V orphan GPR133. Two *Schmidtea *GPR157 homologues (SMD002980 and SMD009091) were also identified via *Adhesion/Secretin *HMM, however, these receptors exhibit vague sequence similarity to more than one GPCR family [33].

Latrophilin-like receptors were found to be present in both flatworms. *S. mediterranea *SMD011811 contains a GPS domain, and can be grouped with sequence fragments SMDC001354A and SMDC001354B. *S. mansoni *SMP176830 contains a Somatomedin B domain, but shares no significant sequence similarity with the identified latrophilin-like planarian receptors. Evidence of the potential druggability of these particular receptors comes from the parasitic nematode *Haemonchus contortus*, where a latrophilin-like receptor has been identified as a target of an anthelmintic cyclodepsipeptide [68]. One other *Adhesion*-like parasite GPCR was identified (SMP058380) that displays an N-terminal GPS domain, but with no clear planarian ortholog.

The *Secretin *flatworm complement is comparatively smaller. Two *S. mansoni *and one *S. mediterranea Secretin *GPCRs were identified. SMP125420 and its planarian ortholog SMD004009 show high sequence similarity to diuretic hormone receptors and contain an N-terminal hormone receptor domain (HRM). These receptors likely play a role in homeostatic regulation. *Schistosoma *SMP170560 exhibits an HRM domain and parathyroid hormone receptor homology. This receptor is likely to have a planarian ortholog, but despite the recognition of a short, nearly identical *Schmidtea *sequence fragment, we were unable to identify the rest of the hypothetical gene within the assembly.

### The *Glutamate *Family

*Glutamate *GPCRs respond to a wide range of signals, including glutamate, *γ*-aminobutyric acid (GABA), Ca^2+ ^and odorants. The mammalian complement of metabotropic glutamate receptors (mGluRs) consists of 8 proteins that fall into three groups. They universally possess a large extracellular domain that contains a ligand binding domain (LBD). The *Drosophila *mGluR-like complement consists of two receptors, DmGluRA and DmXR. DmGluRA shares the structural profile of mammalian mGluR2 and mGluR3. DmXR constitutes one member of a larger insect-specific clade, and displays an atypically-diverged LBD that responds to L-canavanine [69,70]. Outside of the metazoa, a group of 17 *Dictyostelium *GABA_*B*_-like receptors (GrlA-GrlR) have been forwarded as potential evolutionary precursors to mGluRs [71,72].

**Figure 8:**
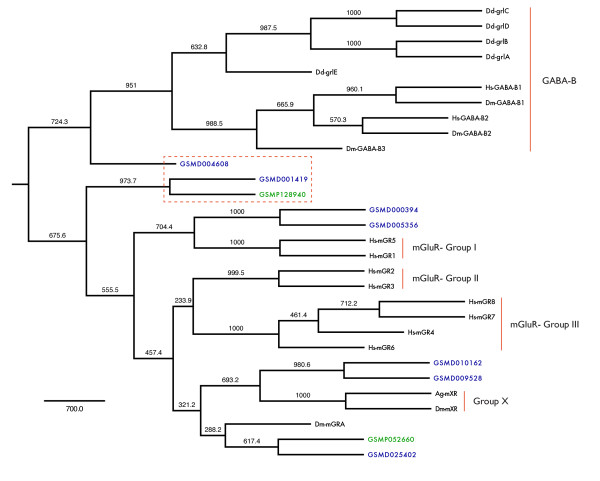
**Phylogenetic analysis of *Glutamate *GPCRs**. Maximum parsimony tree of *Glutamate *family GPCRs. TM domains I-VII were used for phylogenetic analysis with the alignment bootstrapped 1000 times (bootstrap support values are provided). *Schistosoma *sequences are shown in green and *Schmidtea *sequences are shown in blue. GSMD007320 and GSMD015264 were excluded as they remain incomplete over the sequence range used. GABA_*B *_receptors are highlighted, along with the primary vertebrate mGluR groupings and the more recently discovered insect Group X receptors. A human Calcium-sensing receptor (AAA86503.1) was used as an outgroup. Putative flatworm GPCRs that are diverged from both the GABA_*B *_and glutamate-responsive receptors are outlined in red. The ligand-binding domains of these receptors are further analyzed in Figure 9.

**Figure 9:**
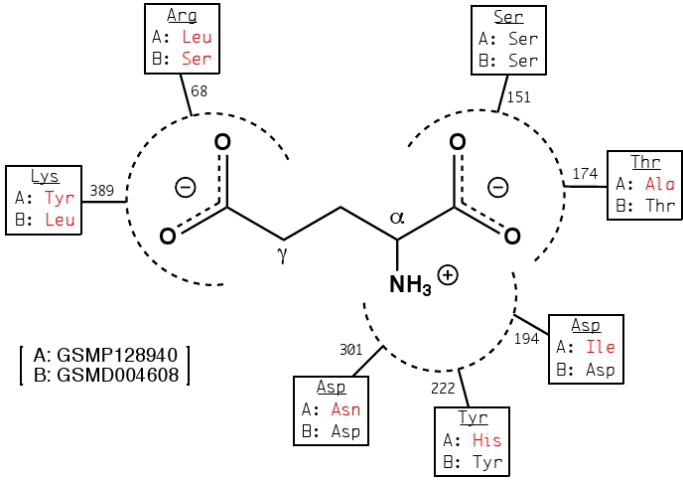
**Schematic of glutamate in association with LBD residues**. Conserved mGluR LBD residues involved in glutamate binding are shown (underlined) in comparison with the corresponding residues for flatworm *Glutamate*-like receptors GSMP128940 and GSMD004608. Numbers represent residue location with respect to the mouse mGluR3 sequence. Disagreement at a given position is highlighted in red. GSMP128940 displays overall divergence with the canonical glutamate binding pocket, while GSMD004608 retains only key residues that interact with the glutamate *α*-carboxylic and *α*-amino groups.

We identified 2 *S. mansoni *and 9 *S. mediterranea Glutamate-like *sequences. Phylogenetic analysis of these sequences was performed with respect to both mammalian and non-mammalian *Glutamate *receptors (Figure 8). The *S. mansoni Glutamate-like *receptors both have corresponding orthologs in the *S. mediterranea *genome. GSMP052660 and its ortholog GSMD025402 group with DmGluRA, and most of the remaining planarian sequences fall in the phylogenetic vicinity of the major mGluR groupings. However, GSMP128940 and its ortholog GSMD001419, along with GSMD004608, seem to be significantly diverged from both GABA_*B *_and mGluR receptors. In the case of DmXR and Grl receptors, the examination of key LBD residues involved in glutamate binding led to the eventually validated conclusion that glutamate was not the primary ligand. We perform similar analysis as depicted in Figure 9. Although the residues of GSMD001419 involved in *α*-amino and *α*-carboxylic groups of glutamate are conserved, the residues associated with the *γ*-carboxylic group are not. This runs parallel to the observations made for DmXR. GSMP128940 displays an even more atypical LBD and conserves only a single putative glutamate-interacting residue. We hypothesize that these particular receptors either bind other amino acid-derived ligands or possess unusual pharmacological profiles.

### The *Frizzled *Family

Wnt-mediated *Frizzled *signaling plays a significant regulatory role in a number of crucial developmental processes, including cell fate determination, cell motility, cell polarity, and synaptic organization [73]. In planaria, the canonical Wnt/*β*-catenin pathway is implicated as a molecular switch for anteroposterior polarity in regeneration [25,74]. We identified four *S. mansoni Frizzled *sequences, along with the 10 *S. mediterranea *sequences previously identified. A single *Smoothened*-like sequence was found for each species.

In humans, 10 *Frizzled *receptors are phylogenetically grouped into four clusters: Fzd1/Fzd2/Fzd7 (I), Fzd5/Fzd8 (II), Fzd4/Fzd9/Fzd10 (III), and Fzd3/Fzd6 (IV) [73]. Both flatworm genomes house a single receptor (FSMP118970 and FSMD000018) that groups in cluster IV, sharing ~45% amino acid identity with Drosophila Fzd1 and ~38% identity with human Fzd6. Four planarian (FSMD023435, FSMD010098, FSMD017743 and FSMD000054) and two schistosome (FSMP139180 and FSMP155340) receptors appear to belong to cluster II. Other flatworm *Frizzled *receptors show less clear relationships with their vertebrate counterparts (Additional File 4).

### Ligand-based support vector machine (SVM) *Rhodopsin *subclassification

Support vector machines (SVM) represent a powerful supervised-learning method for data classification. Given a combined set of positively and negatively labeled training instances, an SVM produces a binary classifier that can then be used to label unknown samples. Each instance is associated with a fixed-length numerical feature vector, containing certain attributes of the data to be classified. The SVM identifies a maximum-margin separating hyperplane to distinguish between vectors representing instances of opposite sign. More often than not, training instances are not linearly separable in the feature space, and feature vectors must first be mapped to a higher dimensional space. Non-linear classification is then performed by application of kernel functions which allow for the construction of a hyperplane in the transformed feature space. Recently, this approach has seen extensive use in the area of biosequence discrimination, and relevant to our goals, the particular problem of GPCR classification.

In the first study on the matter, SVM-based classifiers were shown to drastically outperform their BLAST and HMM-based counterparts for level 1 and level 2 GPCR subclassification [75]. Subsequent studies further improved the predictive performance of SVMs with the introduction of dipeptide composition feature vectors [76,77], achieving accuracies of 97.3% and 96.4% for level 1 (*Rhodopsin*) and level 2 (amine) classification, respectively. Alternative feature vectors have since been similarly validated [78,79]. Although these computational approaches are touted as among the most sensitive, to the best of our knowledge, they have seen no utilization in the realm of genome-wide GPCR mining studies.

Perhaps one reason for this is that even in the case of publicly available SVM classifiers, training and validation occurs exclusively with full-length sequence data. More suitable classifiers would be tailored to the general deficiencies of sequence data resulting from *in silico *methods, where inexact gene structures are an unavoidable phenomenon. In this respect, we developed a classifier to complement our particular GPCR identification approach. This involved focusing SVM training on transmembrane domains, as identification of these conserved blocks had been a primary aim of both our receptor mining and manual curation protocols.

#### Multi-class SVM

Multi-class SVMs refer to classification problems where the number of classes, *k*, is greater than 2. Such problems are typically approached with either the "one-versus-rest" (OvR) or "one-versus-one" (OvO) method. In the OvR scenario, *k *binary classifiers are trained, such that each classifier separates one class from all others. The "winner-takes-all" strategy is then commonly used to label unknown samples, whereby the classifier with the highest output decision function assigns the final class. In the OvO scenario, $\frac{k\left(k-1\right)}{2}$ binary classifiers are constructed in a pair-wise manner. A voting strategy is then typically employed in classification, whereby each classifier accounts for one vote and the class with the maximum number of votes assigns the final label. Although the OvO method has been shown to perform better on a number of fronts [80], as far as the authors are aware, all previously described SVM-based GPCR classifiers available for online use rely on the simpler OvR method. We constructed OvO GPCR classifiers for two levels of *Rhodopsin *family sub-classification.

#### Building feature vectors for ligand-based receptor discrimination

The general fixed-length feature vector, $\vec{F}$, contains frequency information for the 20^2^(400) possible dipeptides over a given stretch of sequence, *L *amino acids in length. Dipeptides are counted in both possible frames and there are therefore *L - *1 total amino acid pairs.

$$\begin{array}{c} \vec{F}=\left\langle P_{1},P_{2},...,P_{399},P_{400}\right\rangle \\ P_{i}=\frac{f_{i}}{L-1},\text{where }f_{i}\text{ represents the frequency of dipeptide }i \end{array}$$

To better associate an SVM-based classification approach with our gene-mining strategy, we explored the idea of again focusing our efforts exclusively on the transmembrane domains. Two options in the way of final feature vector construction were pursued: ${\vec{X}}_{T1}$ and ${\vec{X}}_{T7}$. ${\vec{X}}_{T1}$ represents the 400-element dipeptide frequency vector taken over the entire length of a TM-only pseudosequence, while ${\vec{X}}_{T7}$ represents the 2800-element dipeptide frequency vector generated from the ordered concatenation of the dipeptide frequency vectors for the seven individual TM domains. The standard dipeptide frequency vector calculated for full-length proteins, ${\vec{X}}_{FL}$, was used for comparison. We will refer to the corresponding SVM classifiers as SVM_*T*1_, SVM_*T*7_, and SVM_*FL*_.

$$\begin{array}{c} {\vec{X}}_{T1}={\left\{\vec{F}\right\}}_{TM1-TM7}\\ {\vec{X}}_{T7}={\left\{\vec{F}\right\}}_{TM1}⊕{\left\{\vec{F}\right\}}_{TM2}⊕...⊕{\left\{\vec{F}\right\}}_{TM7}\\ {\vec{X}}_{FL}={\left\{\vec{F}\right\}}_{FL} \end{array}$$

#### SVM training: cross-validation and grid search

*Rhodopsin *training sequences were divided into 17 subfamilies using the GPCRDB classification system. Programs were written to process this training data into feature vector form. Training was performed with the radial basis function (RBF) kernel, $K\left(x_{i},x_{j}\right)=e^{-\gamma ||x_{i}-x_{j}|{|}^{2}}$, and a grid search was used to tune parameters *γ *and *C *with 5-fold cross-validation. For each proposed feature vector construction, the best performing (*C*,*γ*) pair was selected in domains *C *= 2^-5^, 2^-4^, ..., 2^15 ^and *γ *= 2^-15^, 2^-14^, ..., 2^15 ^and used to train a final classifier (Table 4).

**Table 4:** *Rhodopsin *SVM training parameters and cross-validation accuracy

	SVM model	Scoring scheme	*γ*	*C*	5-fold ACC
	SVM_*T*1_	OvO	16.0	32.0	99.01%
Level 2: *Rhodopsin*	SVM_*T*7_	OvO	2^-8^	2048.0	99.47%
	SVM_*FL*_	OvO	256.0	32.0	98.65%

	SVM_*T*1_	OvO	256.0	32.0	96.44%
Level 3: *Amine*	SVM_*T*7_	OvO	32.0	2^4.5^	95.0%
	SVM_*FL*_	OvO	256.0	32.0	94.77%

RBF grid-search parameters used to train SVM models, along with the corresponding 5-fold cross-validation accuracy (ACC) for the training set. The best performing model for level 2 (subfamily) classification is SVM_*T*7_, while the best performing model for level 3 (amine) classification is SVM_*T*1_. Both classifiers exclusively employ transmembrane sequence data, and outperform the classifiers trained with full-length sequence data.

Our original expectation was that SVM_*T*1 _would display lower accuracy than SVM_*FL*_, given that a smaller subset of sequence information would be used for training. We hoped that this presumed disparity would be compensated by SVM_*T*7 _with the addition of position-specific information. Instead, both SVM_*T*1 _and SVM_*T*7 _registered higher cross-validation accuracies than SVM_*FL *_for *Rhodopsin *subfamily classification. ${\vec{X}}_{T7}$ was the best-performing classifier with 99.47% accuracy. These results led us to conclude that for the *Rhodopsin *family, the exclusion of sequence information outside of the transmembrane bundle improves dipeptide-based SVM classification. Encouragingly, this is in agreement with structure and ligand interaction data for the *Rhodopsin *family [81]. The same procedure was carried out in constructing classifiers for amine GPCRs. SVM_*T*1 _was the best performing classifier with a cross-validation accuracy of 96.44%.

#### SVM classification results

*Rhodopsin *sequences with seven TM domains as predicted by HMMTOP were classified by the two-tiered SVM. TOPs were aligned and manually examined to correct for erroneously predicted TM domains. Sequences were then subclassified with the *Rhodopsin *SVM_*T*7 _classifier, and those discerned as amine-responsive were further sub-classified with the amine classifier SVM_*T*1_. A total of 122 *S. mediterranea *and 58 *S. mansoni *sequences were classified via *Rhodopsin *SVM. The majority of these receptors were identified as peptide-responsive (Additional File 5, Table S2). This grouping also contains all PROF1 receptors included in this classification stage, perhaps providing some clues as to their ligands. A subset of 22 *S. mediterranea *and 19 *S. mansoni *sequences were identified as amine-responsive, and classified via amine SVM_*T*1_. These classification outputs are detailed in Table S3 (Additional File 6). These results can inform receptor deorphanization efforts, alongside traditional homology-based approaches.

## Conclusions

This is the first comprehensive genome-wide study of G protein-coupled receptors in the phylum Platyhelminthes. Our transmembrane-focused receptor mining approach yielded a lower-bound estimate of 117 *S. mansoni *and 460 *S. mediterranea *GPCRs. Phylogenetic analysis established the presence of the primary metazoan GRAFS families, along with well-populated *α *and *β Rhodopsin *subfamilies in both examined genomes. The identification of these receptors complements previous and ongoing efforts to identify biogenic amine and neuropeptide-like ligands in flatworms, and will help identify specific receptors that mediate important aspects of flatworm biology associated with the aminergic and peptidergic signaling systems.

The flatworm GPCR repertoire is also shown to house entirely novel receptor groupings with large numerical representation, including a Platyhelminth-specific *Rhodopsin *subfamily (PROF1) and a planarian-specific *Adhesion*-like family (PARF1). These particular lineage-specific expansions, along with the many other highly-diverged and receptors identified, may reveal functional innovations specific to these organisms. Many of these receptors have enhanced appeal as selective pharmacological targets. While their diverged structures are an attractive feature in the parasite drug discovery paradigm, this presents a challenge in posing more exact hypotheses related to receptor function.

To further aid the process of functionally pairing receptors and ligands, we provide a preliminary classification of full-length receptors using SVMs. This represents the first effort to apply SVMs to the problem of GPCR classification in a whole-genome manner, a task made difficult by the evolutionary distance of flatworms from other species with well-characterized GPCR complements. SVM results may be used in conjunction with phylogenetic and homology-based approaches to receptor classification. These results suggest that PROF1 receptors may respond to phylum-specific peptide ligands. As the quality of the underlying gene models improves, and as a greater number of full-length receptor transcripts are sequence characterized, these SVMs can be refined and applied to an expanding subset of identified GPCRs. Functional characterization of flatworm GPCRs is also likely to improve SVM accuracy by providing more relevant training examples.

The notion that schistosome GPCRs represent lucrative anthelmintic drug targets is strengthened by data on the crucial biological role of related receptor signaling molecules in nearly-related organisms [82,83], as well as that of predicted platyhelminth GPCR ligands [56,57,61,63]. The receptors, ligands and downstream biochemical pathways associated with GPCR signaling have been identified as potential targets for parasite life-cycle interruption [23,84]. Enlistment of schistosome reverse genetics approaches alongside receptor sequence data can lead to the validation of specific receptors as drug targets.

In this regard, RNAi in schistosomes [85,86] provides new opportunities for focused exploitation of this dataset. A simple medium-throughput phenotypic classification system has recently been described for both schistosomula and adult schistosomes [87]. These endpoints could readily be used in an RNAi-mediated GPCR loss-of-function screen. Assaying the temporal expression profiles of parasite GPCRs can also be a worthwhile measure as a selection tool for receptors expressed in intra-host stages. On this front, we further the case for planarians as a convenient model organisms to interrogate the function of trematode receptors, and provide a list of inter-species receptor pairings ranked by sequence identity.

While we further the case of planarians as model organisms for flatworm parasite research, differences between the receptor complements of parasitic and free-living flatworms are also very likely to reveal important molecular actors. Phylogenetically distinct planarian receptors are more likely to be involved in regenerative processes, while receptors unique to schistosomes are more likely to play key roles in parasite pathogenesis. Both of these avenues present fertile ground for the targeting and functional elucidation of specific receptors.

## Methods

### Predicted proteomes and training sequences

The most recent release of the *S. mansoni *genomic assembly is accompanied with a set of 13,197 predicted proteins [14]. The *S. mediterranea *predicted proteome consists of 31,955 predicted proteins that were produced with MAKER, although this number may represent a significant overestimate of the true protein count [15,88]. HMM and SVM training sequences were downloaded from GPCRDB [49]. In total, 268 *Glutamate*, 5025 *Rhodopsin*, 175 *Adhesion*, 354 *Frizzled *and 185 *Secretin *sequences were procured for HMM training. 20,920 GPCRDB sequences were used for *Rhodopsin *SVM training, and 2,105 sequences were used for amine SVM subclassification.

### Nomenclature

Putative receptors retain their original GeneDB, MAKER, or GenBlast IDs in modified form. In cases where a gene model was created, receptors were given a label in similar form that includes genomic contig or scaffold information. Letters are appended to the ends of these labels where necessary to distinguish among multiple gene models associated with a single contig or scaffold. All putative flatworm GPCR sequences are provided in association with their tentative IDs (Additional File 7).

### Transmembrane domain prediction

We applied two common algorithms, TMHMM 2.0 [48] and HMMTOP 2.1 [47], to identify transmembrane domains in our GPCR training set. HMMTOP correctly predicted 7 TM domains for 93.8% (4712/5025) of rhodopsin family receptors, compared to 81.9% (4119/5025) in the case of TMHMM. This disparity in sensitivity held for all GPCR families, and was the basis for our decision to employ HMMTOP for most subsequent work. A robust Perl script (Additional File 8) was written to parse coordinate predictions output from HMMTOP, and to generate sequence files containing only regions of interest from the original protein sequences as required.

### TM-focused Profile hidden Markov model (HMM) construction

Provided a multiple sequence alignment, HMMER-2.3.2 [89] builds a probabilistic model (profile HMM) that can be used to query sequence databases to find (or align) homologous sequences. To prepare each GPCR family training set, predicted TM domains flanked bi-directionally by 5 amino acids were extracted and concatenated using coordinates produced in the previous section. These sequences were aligned with Muscle 3.6 [90] and a profile HMM was constructed for each family with *hmmbuild*. All models underwent calibration using *hmmcalibrate*, with the default parameters.

### HMM-based GPCR identification

All predicted proteins in the *S. mansoni *and *S. mediterranea *genomes with a predicted number of TM domains in the range of 3-15 were processed in a manner identical to the HMM training set. These TOP-converted protein sets were searched against our family-specific profile HMMs using *hmmpfam*. The resulting hits for each GPCR family were ranked according to E-value, and a cut-off was selected at the point where subsequent hits showed significant homology to other known proteins or GPCRs belonging to other families. This was accomplished with a BLASTp search of all hits against the NBCI nr database. The BLAST results were parsed with a script and top results were examined for removal of false positives.

### Manual curation of putative GPCR-encoding genes

A large number of GPCR sequences underwent manual inspection of gene structure, and the original predictions were edited where possible. Common manual edits included the merging or splitting of gene models, modification of intron-exon boundaries, and sequence extension or truncation in either or both directions. All editing was performed with Artemis [91]. Curation was primarily guided by homology-based searches and identification of TM domains and family-specific GPCR motifs in ORFs that occurred in the vicinity of a gene model. In the case of *S. mansoni*, this labor-intensive process was aided by the extraction of GeneDB [http://www.genedb.org/] annotations for scaffolds thought to contain one or more receptors. More specifically, a script was written to compile pertinent scaffold information stored in EMBL formatted files, including the orientation, the number of predicted transmembrane domains and the top BLAST hits for proteins identified by the profile HMMs. This data was parsed into a spreadsheet and proved significant in helping identify instances where manual curation was appropriate (Additional File 9). In the case of *S. mediterranea*, annotated genomic regions were loaded into Artemis and edited in a similar manner.

### Phylogenetic analysis

Near full-length (TM > 5) receptors were first processed for removal of the N- and C-termini. ClustalX 2.0 [92] was used to generate multiple sequence alignments of the GPCRs to be examined, with default parameters. PFAAT [93] was used to edit the resulting alignment with attention to key motifs and residues housed within transmembrane domains. Low-entropy sequence blocks present in all sequences were retained. The Phylip 3.6 [94]package was used to generate phylogenetic trees. Alignments were bootstrapped using *seqboot*. Maximum parsimony trees were calculated with *protpars *with input order randomized. Neighbor-joining trees were calculated with *protdist *and *neighbor *using the JTT (Jones-Taylor-Thornton) distance matrix and with input order randomized. Consensus trees were built with *consense*, and visualized and edited with FigTree [http://tree.bio.ed.ac.uk/software/figtree/].

### PROF1 RT-PCR

Total RNA was extracted from flatworm (schistosome or planarian) tissue using the RNAqueous Kit (Ambion), and RNA was treated with Turbo DNAase (Ambion) per manufacturer's instructions. A two-step RT-PCR was performed, where reverse transcription was first carried out with the Retroscript kit (Ambion). Primers were designed for two schistosome PROF1 sequences and 13 planarian PROF1 sequences using Primer 3.0 [95] (Additional File 10). PCR products were visualized by agarose gel electrophoresis to confirm transcript expression.

### SVM

Programs were written to process training sequences into feature vector form for the training of three SVM classifiers: SVM_*T*1_, SVM_*T*2_, and SVM_*FL *_(Additional File 3). TM prediction was performed on training sequences with HMMTOP, and fixed-length dipeptide frequency vectors were calculated in correspondence with each model. SVMs were implemented with the the LIBSVM [96] package. The RBF kernel was chosen and a grid-search was performed with an available python script for selection of kernel parameters. *C *and *γ *were assayed in the domains *C *= 2^-5^, 2^-4^, ..., 2^15 ^and *γ *= 2^-15^, 2^-14^, ..., 2^15 ^to identify the *C*,*γ *pair that maximizes 5-fold cross validation ACC. The classifiers were trained in accordance with the GPCRDB ligand-based groupings, and applied to a subset of flatworm *Rhodopsin *receptors with 7 predicted TM domains.

## Authors' contributions

MZ, MJK, AGM, TAD conceptualized and developed the approach. MZ implemented the bioinformatics protocol and carried out the RT-PCRs. MZ, MJK, PM, and SAC were involved in phylogenetic analysis and interpretation of the results. All authors contributed to the drafting and revision of the manuscript. All authors read and approved the final manuscript.

## Supplementary Material

Additional file 1 **HMM and BLAST filter output**. ZIP archive containing spreadsheet files in excel format: species-specific ranked GRAFS HMM results and BLAST filter results for the *Rhodopsin *family. Click here for file

Additional file 2 **Homology-based annotation of final *Rhodopsin *datasets**. ZIP archive containing spreadsheet files with the top BLAST results for the final *Rhodopsin *receptor datasets ranked by E-value. Tentative BLAST-based classifications are provided as separate text files. Click here for file

Additional file 3 **Table S1. Identification of Planarian sequelogs of parasite *Rhodopsin *receptors**. The nearest *S. mediterranea *sequelog of each *S. mansoni Rhodopsin *receptor is shown, along with the length of the BLASTp overlap region and the corresponding E-value. Receptor pairs are ranked by percent identity (PID). Parasite receptors closest to top of the table are likely candidates for indirect characterization via investigation of their nearest-related planarian counterparts. Click here for file

Additional file 4 **Phylogenetic trees**. ZIP archive containing original consensus trees with bootstrap values and sequence labels in standard NEXUS format. Click here for file

Additional file 5 **Table S2. *Rhodopsin *SVM classifier results**. Ligand-based classification of flatworm *Rhodopsin *GPCRs with *Rhodopsin *SVM_*T*7_. PROF1 receptors are labeled with '*'. A total of 180 receptors were classified, with the vast majority placed in the peptide and amine groupings. Interestingly, all 45 PROF1 receptors were classified as peptide-responsive. Click here for file

Additional file 6 **Table S3. Amine SVM classifier results**. Ligand-based classification of flatworm amine-responsive GPCRs with amine SVM_*T*1_. A total of 41 receptors were identified as aminergic via *Rhodopsin *SVM classification. In cases of erroneous TM boundary prediction, the SVM_*FL *_classifier was used. The classifier results display correct predictions for the three schistosome receptors thus far deorphanized in this subfamily, including two histamine-responsive GPCRs and one dopamine-responsive GPCR (labeled with '*'). Click here for file

Additional file 7 **Receptor protein sequences**. ZIP archive containing all sequences identified, curated, classified, and used in phylogenetic analysis, grouped by family. Click here for file

Additional file 8 **Perl scripts**. ZIP archive containing Perl source code written to perform the work described. Click here for file

Additional file 9 **Parsed schistosome EMBL files**. CSV file containing EMBL parser output. This preliminary output of schistosome auto-annotated GPCR-related receptor genes helped guide first-pass manual curation. Click here for file

Additional file 10 **PROF1 primers**. Text file containing PROF1 primer information. Click here for file
