# Supplementary figures and images for: The repertoire of G protein-coupled receptors in the human parasite Schistosoma mansoni and the model organism Schmidtea mediterranea
Source: BMC Genomics. 2011 Dec 6;12:596. doi: 10.1186/1471-2164-12-596 (PMC3261222; doi:10.1186/1471-2164-12-596)

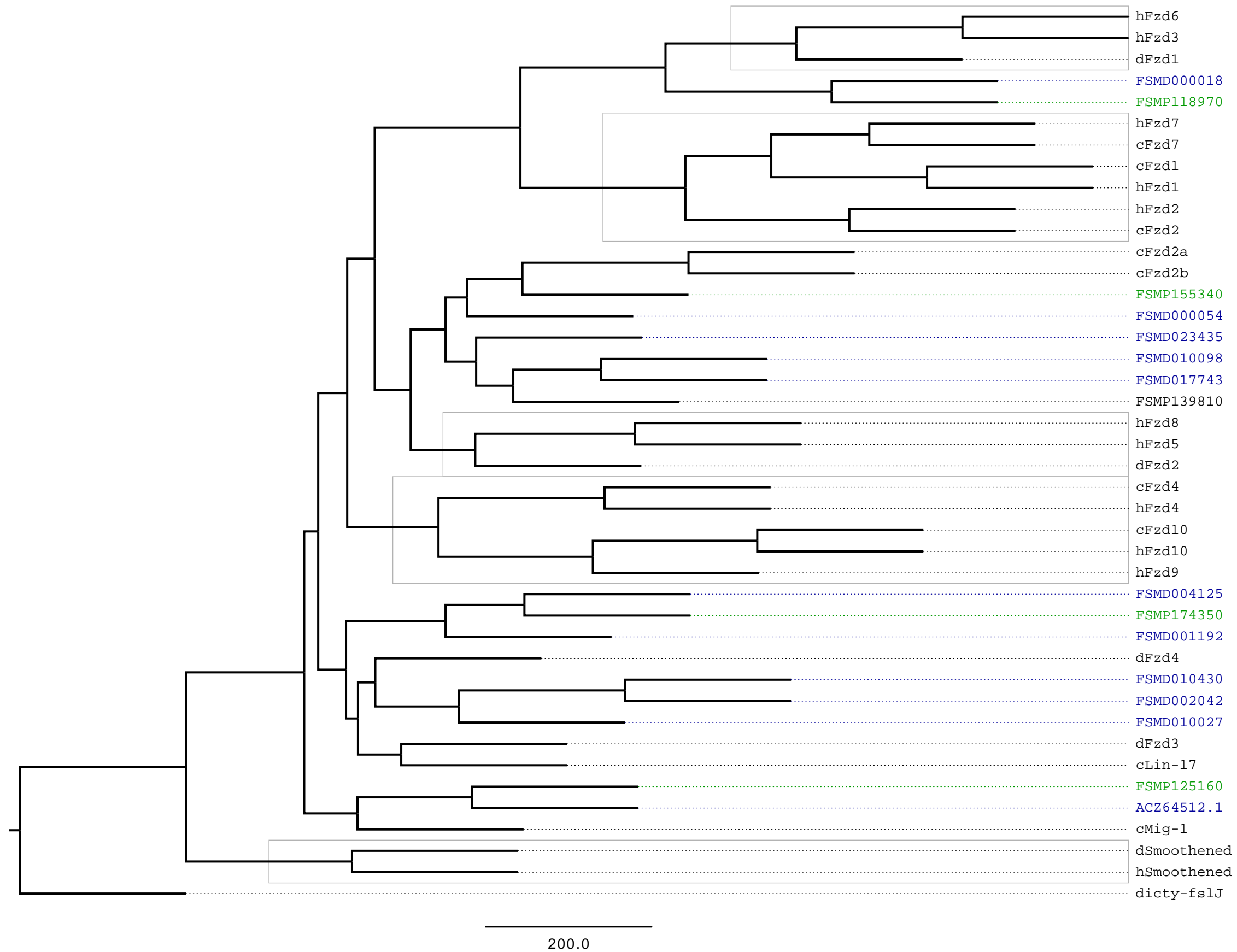

Supplement: Additional file 4 — Phylogenetic trees. ZIP archive containing original consensus trees with bootstrap values and sequence labels in standard NEXUS format. [file 1471-2164-12-596-S4.ZIP › 7-Trees/F-tree.pdf]
